# Extending the Shelf Life of Atlantic Salmon (*Salmo salar*) with Sub-Chilled Storage and Modified Atmosphere Packaging in Recyclable Mono-Material Trays

**DOI:** 10.3390/foods13010019

**Published:** 2023-12-20

**Authors:** Sherry Stephanie Chan, Birgitte Moen, Trond Løvdal, Bjørn Roth, Astrid Nilsson, Marit Kvalvåg Pettersen, Bjørn Tore Rotabakk

**Affiliations:** 1 Department of Processing Technology, Nofima AS, 4021 Stavanger, Norway; sherry.chan@nofima.no (S.S.C.); trond.lovdal@nofima.no (T.L.); bjorn.roth@nofima.no (B.R.); 2 Department of Food Safety and Quality, Nofima AS, 1433 Ås, Norway; birgitte.moen@nofima.no (B.M.); marit.kvalvag.pettersen@nofima.no (M.K.P.); 3 Department of Food and Health, Nofima AS, 1433 Ås, Norway; astrid.nilsson@nofima.no

**Keywords:** sub-chilling, Atlantic salmon, modified atmosphere packaging, shelf life, quality, packaging materials, circular economy

## Abstract

This study investigated the effect of sub-chilling whole gutted salmon and sub-chilled storage at −1 °C in modified-atmosphere packaging in two recyclable mono-material trays (CPET, HDPE). Quality parameters were measured, including water-holding properties, salt content, color, texture, lipid oxidation, and sensory and microbiological shelf life. The oxygen transmission rate was measured for the packages. Compared to traditional fish storage on ice, sub-chilling gave a 0.4% weight gain, better water-holding capacity, and higher salt content. The sub-chilled fish gave a significantly better sensory quality and microbiological shelf life of up to 49 days. Photobacterium was the dominating bacteria during storage. Salmon packaged in CPET trays had a higher drip loss than HDPE trays, but a lower rate of lipid oxidation (1-penten-3-ol). Our results showed the feasibility of significantly extending shelf life with sub-chilling, removing the need for ice. Moreover, using recyclable trays for packaging contributes to a circular economy without compromising food quality.

## 1. Introduction

Food waste is a serious global issue affecting the environment, society, and the economy. The United Nations Sustainable Development Goals aim to halve global food waste by 2030. To prevent food waste, food packaging plays a vital role in protecting food products and prolonging their shelf life [1]. A popular food packaging method for fish is modified-atmosphere packaging (MAP), utilizing various gas compositions in the headspace before sealing the package to delay microbial growth and unwanted chemical reactions. The most used gases for MAP are CO_2_, O_2_, and N_2_, and the gas compositions highly depend on the fish species. For example, fatty fish, such as salmon, are more susceptible to lipid oxidation and require a significantly lower amount of O_2_. In MAP, CO_2_ is the most crucial gas due to its bacteriostatic and fungistatic properties [2,3]. It is water- and lipid-soluble and dissolves easily into food products to inhibit bacterial growth, extending the lag phase and slowing the growth phase of microorganisms [4].

The absorption of CO_2_ into the product can, however, lead to pressure or volume reduction in the package, resulting in package collapse and increased drip loss. Therefore, N_2_ is often added as a filler or balance gas. The effectiveness of MAP is dependent on many factors, including raw material quality, handling and hygiene, gas-to-product (g/p) volume ratio, gas mixtures, storage temperature, and properties of the packaging material [5]. For instance, a high microbial load before product packaging will significantly shorten the shelf life regardless of whether the product is packaged in MA. Therefore, an initial low microbial load is necessary to utilize the benefits of MAP. The gas around the food product in MAP protects against quality deterioration and acts as a buffer for temperature control. The solubility of CO_2_ in the liquid phase increases with decreasing temperature and increasing pressure [6]. As a result, a high degree of filling is needed, which requires higher CO_2_ partial pressure for an equal amount of CO_2_ to be dissolved into the product. For seafood packaging, a g/p ratio between 2 and 3 is usually recommended to avoid package collapse and safeguard the bacteriostatic effects of CO_2_ [2].

The efficacy of the food packaging material and its ability to retain a product’s quality highly depends on its barrier properties. For highly perishable products, such as seafood, the permeability of gases and water vapor of the material is especially important. Laminates or multi-layered materials for food packaging often offer good gas barriers and mechanical properties. However, such materials are made of various substances and are challenging for mechanical recycling. Therefore, they usually end up in incineration or landfills [7]. On the other hand, mono materials can potentially be used to contain seafood in MA. Although mono materials such as polyethylene terephthalate (PET) and polyethylene (PE) are also derived from fossil-based sources, they can be mechanically recycled to make new products, contributing to a more circular economy [8]. There is currently limited research using mono-material trays for MAP on seafood. This research area is of interest to replace conventional packaging materials in the future, as long as it does not compromise the product’s quality and shelf life.

Super-chilling is a food conservation method that lowers the product’s internal temperature to below 0 °C between conventional chilling and freezing [9]. The term “super-chilling” has been interchangeably used with terms like “supercooling”, “sub-chilling”, “deep chilling”, “ultra-chilling”, “surface freezing”, and “partial freezing” [10,11]. This definition is currently not explicitly defined in the legislation [11], although several studies define super-chilling as lowering the internal temperature 1–2 °C below the freezing point of the product, with a certain degree of ice crystal formation [11,12,13,14]. In this study, our focus is to lower the product’s internal temperature to that below melting ice down to the initial freezing point, with no formation of ice crystals. Hence the term “sub-chilling” is used.

An efficient way of sub-chilling bulk volumes of whole fish is using refrigerated seawater (RSW), commonly used in the pelagic industry [15]. Recent studies have shown that sub-chilling whole gutted fish in RSW at −1 °C gives a higher water-holding capacity and comparable quality to storing on ice [16,17]. However, using ice for storage can substantially occupy up to 30% of the weight, contributing to extra costs and logistical challenges [18,19]. The concept of hurdle technology combines preservation techniques to reduce or inhibit microbial growth. As part of a hurdle technology strategy, the sub-chilling of salmon can be combined with packaging methods such as vacuum packaging [20,21] and MAP [22]. Sivertsvik, Rosnes, and Kleiberg [22] evaluated the effect of MAP with super-chilled storage at −2 °C on salmon fillets and reported a better microbiological and sensory shelf life than with those stored at chilled conditions (4 °C). The study showed the potential of extending shelf life to more than 24 days. Therefore, this study combines recyclable mono-material trays to evaluate the synergistic effect of sub-chilling whole gutted Atlantic salmon in RSW and sub-chilled storage in MAP after processing.

## 2. Materials and Methods

### 2.1. Experimental Design

**Figure 1:**
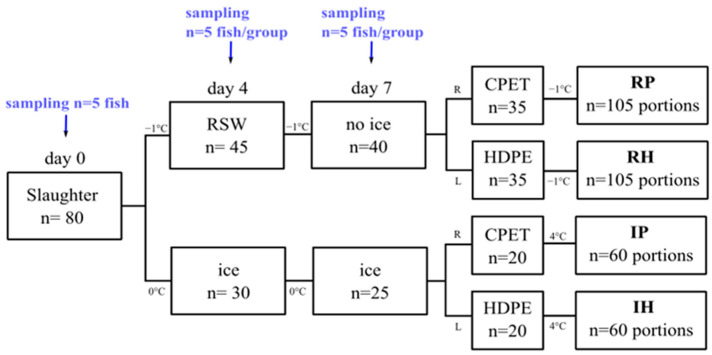
Graphical illustration of the experimental setup. RSW and ice represent whole storage in RSW or ice, respectively. R and L represent right and left fillets. RP and RH represent portions where whole fish were stored in RSW before MAP in CPET or HDPE trays. IP and IH represent portions where whole fish were stored on ice before MAP in CPET or HDPE trays.

Eighty gutted Atlantic salmon were obtained from a nearby slaughter facility in March 2022 (starved for 14 days; average weight: 4.51 ± 0.3 kg). These fish were split into 2 groups, where 10 were tagged and weighed for each group to monitor the weight change for 7 days. One group was packaged in expanded polystyrene (EPS) boxes with ice (*n* = 30) and kept in a 0 °C cold room. The other group (*n =* 45) was immersed in a 1000-L polyethylene tank containing salt water made in the laboratory (3.5% salinity) for 4 days, before the salt water was drained and the fish placed in EPS boxes without ice for 3 days at −1 °C. The temperature of the salt water was maintained at −1 °C by the addition of pre-made saltwater ice, in accordance with Chan et al. (2021). Wireless temperature loggers (TrackSense Pro, Ellab A/S, Hillerød, Denmark) were placed in random fish from each group throughout the experiment. Sampling was carried out periodically (*n =* 5/group) on days 0, 4, and 7 *post-mortem* for pH, drip loss, water-holding capacity, color, texture, salt, and microbiological analysis. In addition, 3 extra fillets from day 0 were used to measure the freezing point with the insertion of temperature loggers (Testo 176T4, Max Sievert AS, Oslo, Norway), and the fillets were stored in a −30 °C freezer (GLE50, Frigor, Viborg, Denmark). A graphical illustration of the experimental setup is shown in Figure 1.

### 2.2. Processing and Packaging

Fish were divided into 2 main groups according to how the whole fish was stored (R: RSW, I: ice). On day 7, the remaining fish were manually filleted and further split into two groups based on the type of tray used for MAP (P: CPET, H: HDPE). This results in a total of 4 groups (RP, RH, IP, IH), as illustrated in Figure 1. The right and left fillets were portioned into 3 portions per fillet (portions A, B, and C; *n =* 105 portions/group).

As mentioned, two types of mono-material packaging materials in trays were applied in both storage methods after portioning and MAP (60% CO_2_:40% N_2_ gas mixture; gas-to-product (g/p) ratio: 2:1), a crystallized polyethylene terephthalate (CPET) tray (C2187-F black, 680 mL, Færch, Holstebro, Denmark) versus a high-density polyethylene (HDPE) tray (Dyno 513, 550 mL, Berry Packaging, Kristiansand, Norway). A Multivac T200 Tray sealer (Multivac, Wolfertschwenden, Germany) was used to seal the CPET trays with a PET sealant (Cryovac OSF33ZA, thickness 33 µm, oxygen permeability 60 cm^3^/m^2^/24 h/bar (23 °C, 0% RH), achieving a water vapor transmission rate 22 g/m^2^/24 h (38 °C, 100% RH, Sealed Air, Rudshøgda, Norway). Simultaneously, a semiautomatic Dynopack tray sealer (type VGA 462, Berry Packaging, Norway) was used to seal the HDPE trays with polyamide/low medium-density polyethylene (Dynoseal GPO 1570, 15 µm OPA/70 µm LMDPE) top web (Berry Packaging, Kristiansand, Norway). To obtain a g/p ratio of 2, fillets were portioned to around 230 g and 180 g per piece for packaging in CPET and HDPE trays, respectively. RP and RH were stored at sub-chilled conditions at −0.7 ± 0.4 °C, while IP and IH were stored at chilled conditions at 4.4 ± 0.4 °C.

### 2.3. Quality Analysis

**Figure 2:**
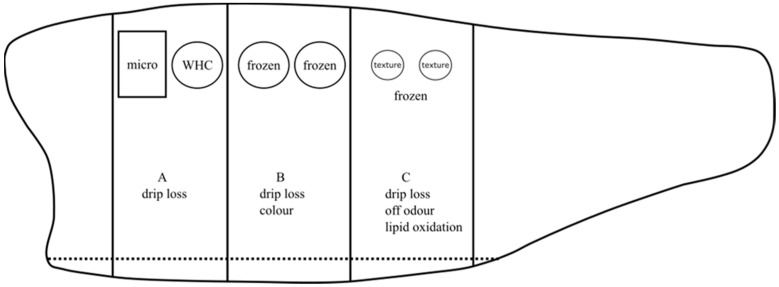
A graphical illustration of fillet portions A, B, and C from the same fillet, where quality analyses were performed in each portion. “Micro” and “WHC” indicate microbiology and water-holding capacity samples, respectively. “Frozen” in portions B and C indicates samples frozen at −80 °C for further analyses of salt content and lipid oxidation, respectively.

After portioning and packaging on day 7, quality analysis was carried out periodically 11, 14, 18, and 21 days after slaughter for the iced (IP and IH) group. For the RSW (RP and RH) group, quality analysis was carried out 14, 21, 29, 35, 42, 49, and 56 days after slaughter. On each sampling day, portions A, B, and C originating from the same fish were used (Figure 2). Drip loss was measured on all portions. In addition, microbiology and water-holding capacity (WHC) samples were measured in portion A. For portion B, color measurements were performed, and samples were obtained and frozen at −80 °C for further analysis of salt content. Lastly, off-odor, lipid oxidation and textural analysis were carried out on portion C. Samples for analysis of volatile lipid oxidation products were wrapped in aluminum foils and stored at −80 °C for GCMS headspace analysis.

#### 2.3.1. Headspace Gas and Oxygen Transmission Rate

The headspace composition of the packages was analyzed using a PBI Dansensor CheckMate 9900 Headspace gas Analyzer (Nordic Supply System, Skodje, Norway) during each sampling day for % CO_2_ and % O_2_ levels (*n =* 15/group). The oxygen transmission rates (OTR) of empty CPET and HDPE trays sealed with their respective top webs and filled with 100% N_2_ gas and 10 mL water were measured at −1 °C and 4 °C (*n =* 5/group). The ambient oxygen ingress rate (AOIR) method [23] was used.

#### 2.3.2. Drip Loss and Water-Holding Properties

Weight changes of the whole fish were measured on days 4 and 7 (*n =* 10/group). Drip loss of the packaged portions was immediately measured after the packages were opened (*n =* 15/group) and was calculated based on the % weight change with respect to the initial weight. Water-holding capacity (WHC) was analyzed using the method described by Skipnes, et al. [24], including the water content in the calculation (*n =* 5/group). Muscle samples for WHC were obtained using a metal cylinder (diameter 31 mm). The samples were transversally sliced into 2 pieces, where the top piece was used for low-speed centrifugation at 1800 rpm (15 min, 4 °C). The bottom piece was used for water content measurement via the drying of the sample at 105 °C for 18 h.

#### 2.3.3. Salt Content

The salt content from frozen samples in portion B (Figure 2) on days 0, 4, 7, and 49 was analyzed with an EasyCl titration system (Mettler Toledo, Oslo, Norway) (*n =* 5/group). The titration agent used was 0.1 M AgNO_3_. An amount of 100 mL of warm deionized water was added into around 1.5 g of sample. The mixture was then homogenized for 40 s at 13,500 rpm using an Ultra Turrax T25 (Janke & Kunkel IKA, Labortechnik, Staufen, Germany). An amount of 1 M HNO_3_ was added to the resulting mixture, and automatic titration was carried out. Titration stopped until the equivalence point was reached, where AgCl was formed. The % salt was calculated based on the formula (Eq − B) × T × M × F1/W, where Eq is the volume (mL) of AgNO_3_ used at equivalence point; B = 0, blank value; T = 0.1 mol/L, the concentration of titrant; M = 58.44 g/mol, the molecular weight of NaCl; F1 = 0.1, the conversion factor for % and W = sample weight (g).

#### 2.3.4. Color and Texture

Color analysis was carried out using the digital imaging system (DigiEye full system, VeriVide Ltd., Leicester, UK) connected to the digital camera (Nikon D80, 35 mm lens, Nikon Corp., Tokyo, Japan) (*n =* 5/group). The software Digipix v2.8 (VeriVide Ltd., Leicester, UK) was used to calculate the L*a*b* values obtained from the images, where L*, a*, and b* represent lightness, redness, (a* > 0) and yellowness (b* > 0), respectively [25].

Textural analysis was performed using a texture analyzer TA-XT^®^ plus (Stable Micro Systems, Godalming, UK) with a 50 kg load cell (*n =* 5/group) (modified method after Lerfall et al. [26]). A flat-end cylinder probe (12.7 mm P/0.5) was used at a rate of 2 mm/s until the probe reached 80% of the fillet height. Analysis was carried out in duplicates per sample. The force-time graph was recorded using the Texture Exponent software version 8.0.16.0 (Stable Micro Systems, Godalming, UK).

#### 2.3.5. Sensory—Off Odor

A semi-trained internal panel of 4 to 5 assessors conducted the sensory evaluation (*n =* 5/group). Portion C samples were re-packaged in plastic pouches (PA/PE, 160 × 200 mm, LietPak, Čekoniškės, Lithuania) and marked with a randomized 3-digit code before acclimatizing at room temperature for at least 15 min. The evaluation was selected based on the odor category from the fillet index method [27], where the criteria for odor were graded on a 4-point scale (0: best, 3: worst). Samples were deemed spoiled with an average score of above 2.

#### 2.3.6. Lipid Oxidation

Dynamic headspace–Gas Chromatography Mass Spectrometry (GCMS) of volatile oxidation products, as described by Olsen, et al. [28], was used with minor modifications for studying possible lipid oxidation in fish filets. Samples (*n =* 2/group) from the day 0 and day 2 random samples from the RH and RP group were used for the analysis on day 49. They were homogenized before samples of 10 g were weighed into an Erlenmeyer bottle, and ethyl heptanoate in methanol was added as an internal standard. The samples were then placed in a water bath at 70 °C and purged for 30 min with Nitrogen (100 mL/min). Volatile compounds were trapped on an adsorber (Tenax GR, Scientific Instrument Services, Ringoes, NY, USA), desorbed at 280 °C for 5 min in a Markes Thermal Desorber and transferred to an Agilent 6890 GC with an Agilent 5973 Mass Selective Detector (EI, 70 eV) (Agilent Technologies, Wilmington, DE, USA). The volatiles were separated on a DB-WAXetr column (30 m, 0.25 mm i.d., 0.5 μm film) with a temperature program starting at 30 °C for 10 min, increasing 1 °C/min to 40 °C, 3 °C/min to 70 °C, and 6.5 °C/min to 230 °C, with a hold time of 5 min. The peaks were integrated, and compounds were tentatively identified with HP Chemstation software (v. G1701 E.02.02.SP2) and NIST11 Mass Spectral Library. System performance was checked with blanks and standard samples before and after analysis. The samples were analyzed in duplicates.

#### 2.3.7. Microbiological Analysis

Microbiological analyses were determined on every sampling day using the NMKL method No. 184 [29] for total aerobic psychrotrophic counts (TPC), total aerobic mesophilic counts (TMC), and hydrogen sulphide-producing bacterial counts (HSPB) (*n =* 5/group). Around 10 g of muscle samples without skin were homogenized with 90 mL sterile saline water (0.85 g/100 mL) in a Smasher^®^ (AES Laboratorie, bioMérieux Industry, St. Louis, MO, USA) for 2 min. About 50 mL of the stomacher solution was transferred to Falcon tubes and kept in cold storage for one day before pelleting for gene sequencing, as outlined below. Appropriate dilution series were made, and 49.2 µL were transferred to Long and Hammer (L&H) plates using an Eddy Jet 2 W Spiral Plater (IUL micro, Barcelona, Spain) to quantify for TPC. In addition, 1 mL was transferred to iron agar (IA) plates supplemented with 0.04% L-cysteine (Sigma Aldrich, Oslo, Norway) to quantify TMC and HSPB by counting the total and black colonies, respectively. The L&H plates were incubated at 15 °C for 5 days, while iron agar (IA) plates were incubated at 25 °C for 72 ± 6 h. Microbial concentrations are expressed as log cfu/g.

#### 2.3.8. DNA Extraction and Illumina Partial 16S rRNA Gene Sequencing

Samples from days 14, 21, and 49 were chosen for Illumina partial 16S rRNA gene sequencing (microbiota analysis) (*n =* 5/group). Each stomacher solution from the tubes was centrifuged (Heraeus Multifuge X3 FR, VWR International AS, Oslo, Norway) using a fixed angle rotor (Fiberlite F15-8x50cy Rotor, 261× *g*, 5 min, 4 °C, Thermo Scientific, Oslo, Norway). The suspension was filtered using a Whatman 589/1 filter paper (Whatman, Maidstone, UK), and the supernatant was collected in a 50 mL Falcon tube. The tubes were re-centrifuged at 6534× *g*, 20 min, 4 °C. The supernatant was discarded, and the remaining pellet was resuspended in 1.5 mL sterile deionized water. The final suspension was transferred to 2 mL Eppendorf tubes, centrifuged at 12,225× *g* for 2 min (Eppendorf MiniSpin, Eppendorf Norway AS, Oslo, Norway) at room temperature before the supernatant was discarded and the tubes containing pellets were stored at −80 °C for microbiota analysis.

Following the kit protocol, DNA extraction from pellets was carried out with the DNeasy PowerLyser PowerSoil kit (Qiagen, Hilden, Germany). The pellets were homogenized by tissue homogenizer (Precellys Evolution 24 Tissue homogenizer) for 3× 40 s at 7400× *g* rpm with 10 s between intervals.

The 16S rRNA gene PCR (V4 region) and paired-end sequencing (2 × 150 bp) using the MiSeq Reagent Kit v3 on a MiSeq instrument (Precellys Evolution 24 Tissue homogenizer, Bertin Instruments, Bertin Technologies SAS, France Illumina, San Diego, CA, USA) was performed using the protocol presented by Caporaso, et al. [30] as previously described [31]. The sequences were processed in QIIME2 (qiime2-2020.11) [32]. Briefly, the data were demultiplexed using demux; the paired ends were joined using vsearch, quality filtered based on a q-score above 30, and denoised using deblur 16S; and taxonomy was achieved using classify-sklearn with the SILVA database [33,34,35,36,37,38,39]. Singletons were removed, and taxa were collapsed to level 6 (genus level). The taxonomy and feature table were exported to text files and processed in Excel. The feature table was converted to relative values, and taxa below an average of 0.02% across all samples were represented as “Other”. To obtain an estimate of the microbial development of the dominating bacteria (taxa above 0.02%) during storage, the log of the relative abundance values multiplied with the total aerobic psychotropic counts (log10 (relative abundance × CFU/g), was calculated.

#### 2.3.9. Statistical Analysis

Statistical analysis was performed in Minitab^®^ version 21.1.1 (Minitab, Coventry, UK). A general linear model was used, where the chilling method (RSW/ice) was set as a categorical factor, and storage days were the continuous independent variable. After packaging, the packaging trays (CPET/HDPE) were added as an additional categorical factor. The interaction effect between the chilling method and packaging trays was also included when this was significant. The positions of the fillet portions and the sensory panel were included as additional categorical factors for drip loss and sensory analysis, respectively. For texture analysis, fillet height was incorporated as a covariate. In addition, a *t*-test was carried out for the water-holding capacity results between groups for day 7. All results are presented as mean ± standard deviation. The α-value was set to 0.05.

## 3. Results and Discussion

### 3.1. Temperature

**Figure 3:**
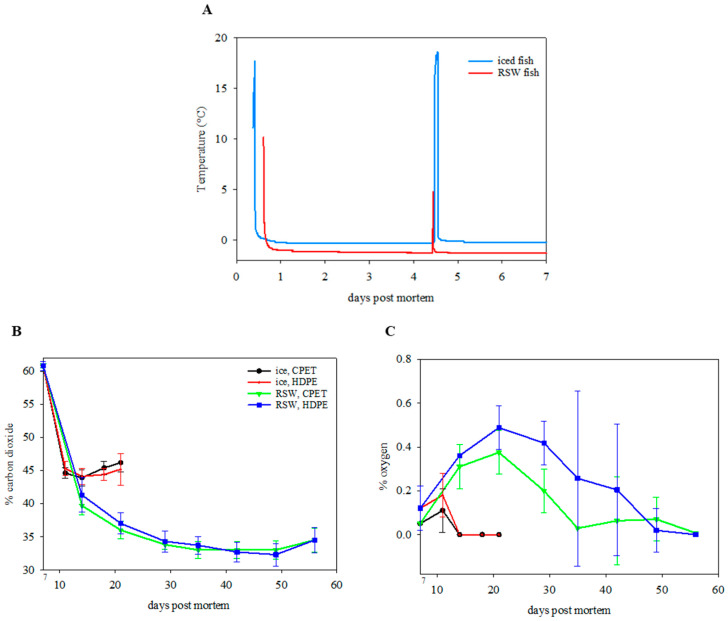
(**A**) Temperature profile of ice- and RSW-stored fish during the initial 7-day storage period. (**B**) Carbon dioxide level (%) (*n =* 18; GLM; storage day: *p* < 0.001; chilling: *p* < 0.001; packaging: *p* = 0.500) and (**C**) oxygen level (%) (*n =* 18; GLM; storage day: *p* < 0.001; chilling: *p* < 0.001; packaging: *p* = 0.001; chilling x packaging: *p* = 0.029) of packaged portions on IP (ice, CPET) and IH (ice, HDPE) for 21 days, and RP (RSW, CPET) and RH (RSW, HDPE) for 56 days.

The freezing point of the raw material was measured as −1.3 ± 0.2 °C. This indicates that phase transition had not yet occurred, and ice crystals did not begin to form in the muscle when they were stored at −1 °C in RSW, giving a fresh product. Figure 3A presents the RSW- and ice-stored fish temperature during the 7 days of whole fish storage. The temperature of ice- and RSW-stored fish remained stable at 0 °C and −1 °C throughout, despite fish being taken out from the RSW on day 4 and placed in EPS boxes. The observed spike in temperature on day 4 was due to random fish being collected for sampling. After filleting and packaging, the RSW-stored fish were maintained at a stable temperature at −0.7 ± 0.1 °C during sub-chilled storage at a −0.7 ± 0.4 °C storage room, while the ice-stored fish were stored at a 3.9 ± 2.4 °C storage room. Temperature is a crucial factor during sub-chilling. A recent study by Cui, et al. [40] reported that a temperature fluctuation of ±2 °C during a 15- and 30-day superchilled storage at −3.5 °C for salmon could lead to higher microbial growth and higher total volatile base nitrogen and lipid oxidation in addition to greater destruction of muscle structure and increased water loss. Maintaining an even temperature throughout the sub-chilling process and storage can be challenging but is feasible, since several actors are involved in the logistical chain, and ice is not present as a temperature buffer.

### 3.2. Oxygen Transmission Rate and Headspace Gas

The OTR for HDPE trays sealed with a laminate film at storage temperatures of −1 and 4 °C were 0.8 ± 0.1 and 1.2 ± 0.1 mL O_2_/(pkg*day), respectively. On the other hand, the OTR for CPET trays sealed with a PET film at −1 and 4 °C were 0.6 ± 0.0 and 0.7 ± 0.0 mL O_2_/(pkg*day), respectively. The AOIR method showed that oxygen diffusion increased as storage temperature increased from −1 to 4 °C (*p* < 0.001). This was expected, as the storage temperature affects the permeability of the packaging material and the diffusion rate of gases, as quantified by the Arrhenius equation. In addition, HDPE trays had a higher OTR than CPET trays (*p* < 0.001), as also shown by Bastarrachea, et al. [41].

Immediately after packaging for all samples, the gas composition was 60.8 ± 0.7% CO_2_ (the rest N_2_), with residual oxygen at 0.1 ± 0.0%. The CO_2_ content decreased rapidly after packaging (Figure 3B). On day 14, the CO_2_ composition decreased to 43.9 ± 1.2% and 44.1 ± 1.2% for the IP and IH groups, with no residual oxygen left. This was followed by an increase in CO_2_ level of 46.2 ± 1.4% (IP) and 45.2 ± 2.4% (IH) on day 21 (*p* < 0.001). For the RSW fish, the O_2_ level peaked at day 21 before gradually decreasing towards the end of storage (Figure 3C). Furthermore, it was observed that the CO_2_ level was lower (*p* < 0.001), while the O_2_ level was higher (*p* < 0.001) for the samples kept at lower temperatures in RSW than on ice. The products packed in HPDE trays gave a significantly higher O_2_ level than the CPET trays inside the packages through storage time (*p* = 0.001) and an interaction effect between the chilling method and the packaging tray (*p* = 0.029).

CO_2_ is more soluble in food products at lower temperatures [6], which explains the decreased CO_2_ composition for the RSW fish stored in sub-chilled storage at −1 °C, as compared to 4 °C for the iced fish. The observed decrease in O_2_ level, in contrast to an increase in CO_2_ level towards the end of storage, could indicate aerobic respiration from the microbial community present in the samples, also correlated to microbial growth.

### 3.3. Water-Holding Properties

**Figure 4:**
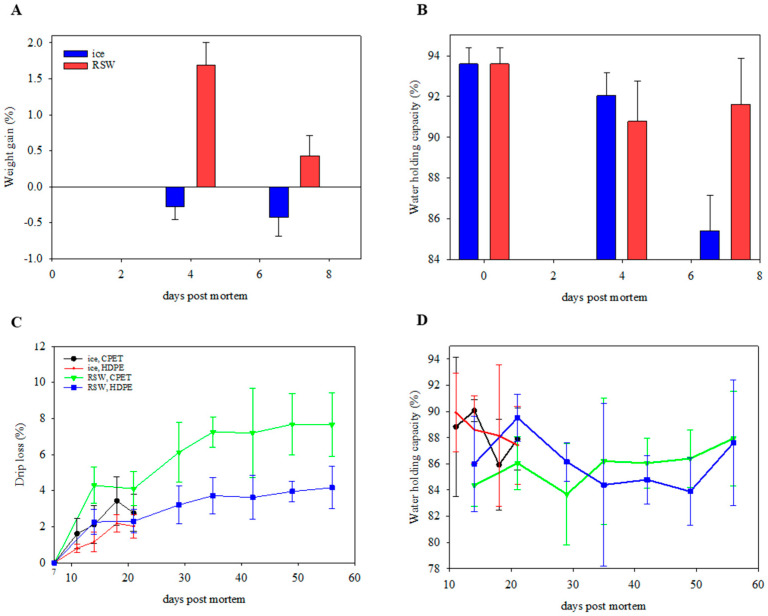
(**A**) Weight gain (*n =* 10; GLM; storage day: *p* < 0.001; chilling: *p* < 0.001) and (**B**) water-holding capacity (*n =* 10; GLM; storage day: *p* < 0.001; chilling: *p* = 0.205) of ice- and RSW-stored fish during the initial 7-day storage period. (**C**) Drip loss (*n =* 18; GLM; storage day: *p* < 0.001; chilling: *p* < 0.001; packaging: *p* < 0.001; position of portion in fillet: *p* = 0.040; chilling × packaging: *p* < 0.001) and (**D**) water-holding capacity (*n =* 18; GLM; storage day: *p* = 0.594 chilling: *p* = 0.019; packaging: *p* = 0.968) of packaged portions on IP (ice, CPET) and IH (ice, HDPE) for 21 days, and RP (RSW, CPET) and RH (RSW, HDPE) for 56 days.

There was a weight gain of 1.7 ± 0.3% for gutted salmon stored for 4 days in RSW (Figure 4A). However, a weight loss of 1.3% was observed when fish were stored sub-chilled in boxes for a further 3 days. Nevertheless, an overall weight gain of 0.4 ± 0.3% was observed (*p* < 0.001). In contrast, the ice-stored fish resulted in a weight loss of 0.4 ± 0.3% after 7 days of storage. Water-holding capacity (WHC) generally decreased through the 7-day storage for both groups (*p* < 0.001). On day 7, the RSW fish had a significantly higher WHC (91.6 ± 2.3%; *p* < 0.001) than the iced group (85.4% ± 1.7%; Figure 4B). These observations were similar to Chan, Roth, Jessen, Løvdal, Jakobsen, and Lerfall [16] and Chan, Rotabakk, Løvdal, Lerfall, and Roth [17], with a weight increase of 1.1–1.6% for fish stored in RSW for 4 days. The higher WHC for RSW fish, particularly on day 7, could be explained by the salting-in process caused by the dissociation of salt ions that contributes to muscle swelling and water retention. This is supported by the significant increase in the salt content of RSW fish from an initial value of 0.16 ± 0.02 to 0.26 ± 0.05% after 7 days (*p* = 0.003). On day 49, the final salt contents of RP and RH fish were 0.31 ± 0.03% and 0.33 ± 0.02%, respectively.

After packaging, drip loss increased over storage time for all groups (Figure 4C, *p* < 0.001). No significant differences were observed between storage days (*p* = 0.594) and packaging trays (*p* = 0.968) on WHC (Figure 4D). However, the WHCs of RSW-stored fish were generally lower (*p* = 0.019), while drip loss was higher (*p* < 0.001) than those on ice after packaging. This could be due to the significantly longer storage duration for RSW fish throughout the study. The position of the fillet portion was also significant, with portion B showing a higher drip loss than portions A and C (*p* = 0.040). An interaction effect was observed between the chilling method and the packaging tray (*p* < 0.001). Those packaged in CPET (RP and IP) had a significantly higher drip loss than their counterparts (RH and IH; *p* < 0.001). A possible explanation is the difference in mechanical properties of the two materials. The elongation at break of PET trays was lower than HDPE, and therefore less flexible. In addition, there was a difference in the packaging trays’ heights, as the CPET trays were shorter than the HDPE trays. This led to the top film touching the salmon portion, which may have applied more physical pressure and influenced the drip loss. The size of the tray may therefore be of importance. This means the packaging volume must be large enough to fill in the required gas and product, resulting in more packaging material. As CO_2_ dissolves into the product, the gas volume reduction can lead to package collapse, giving a “snug down” effect, negatively influencing the sensory appeal and drip loss [7,42,43,44].

### 3.4. Colour and Texture

As presented in Table 1, redness (*p* < 0.001) and yellowness (*p* = 0.005) increased until day 7 before portioning and packaging. Lightness initially decreased from day 0 to 4 and increased on day 7 (*p* < 0.001). For textural properties, the breaking force (*p* < 0.001) and firmness (*p* < 0.001) decreased through storage. The fillet thickness also significantly influenced the breaking force (*p* = 0.002). Table 2 shows packaged portions’ color and textural changes after filleting. Storage days had a significant effect on lightness (*p* < 0.001), redness (*p* = 0.016), and yellowness values (*p* = 0.004). The chilling method did not influence color properties. Those packaged in HDPE trays were generally lighter (*p* < 0.001) and less reddish (*p* < 0.001) in color than fish stored in CPET trays, regardless of the storage method.

Breaking force (*p* < 0.001) and firmness (*p* < 0.001) generally decreased through storage, a known phenomenon of autolytic enzyme activities and protein degradation. This can also influence the reflective properties and color of the muscle [45,46]. Those packaged in HDPE trays had a significantly lower breaking force than in CPET trays (*p* = 0.020). Otherwise, no differences in textural properties between the chilling method and the packaging tray were observed. As expected, fillet height also influenced the samples’ breaking force (*p* = 0.001) and firmness (*p* = 0.012).

### 3.5. Sensory and Microbiology Shelf Life

**Figure 5:**
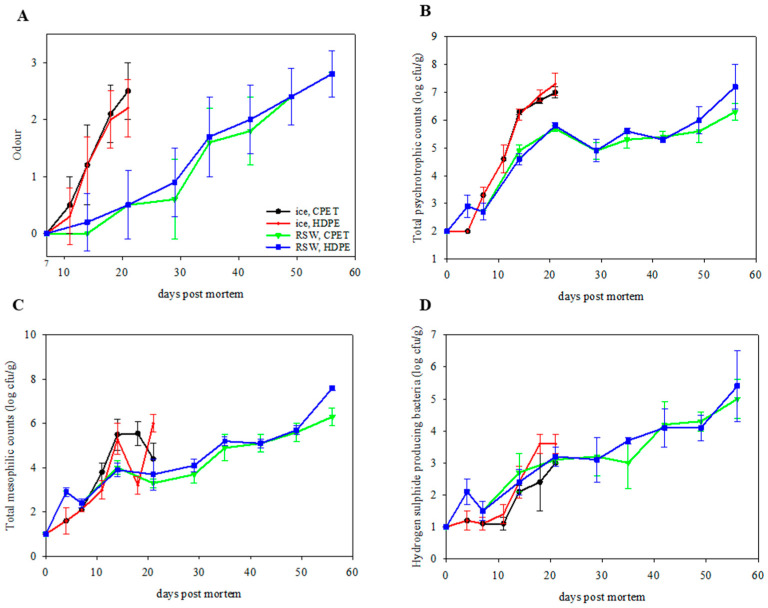
(**A**) Off-odor evaluation of packaged portions (*n =* 6; GLM; storage day: *p* < 0.001; chilling: *p* < 0.001; packaging: *p* = 0.976; judge: *p* < 0.001; chilling x packaging: *p* = 0.048) on IP (ice, CPET) and IH (ice, HDPE) for 21 days, and RP (RSW, CPET) and RH (RSW, HDPE) for 56 days. (**B**) Total psychrotrophic counts (*n =* 6; GLM; storage day: *p* < 0.001; chilling: *p* < 0.001; packaging: *p* = 0.174), (**C**) total mesophilic counts (*n =* 6; GLM; storage day: *p* < 0.001; chilling: *p* < 0.001; packaging: *p* = 0.073), and (**D**) hydrogen producing sulphide bacterial counts (*n =* 6; GLM; storage day: *p* < 0.001; chilling: *p* = 0.535; packaging: *p* = 0.006; chilling × packaging: *p* = 0.048) of packaged portions on IP (ice, CPET) and IH (ice, HDPE) for 21 days, and RP (RSW, CPET) and RH (RSW, HDPE) for 56 days.

The sensory off-odor analysis indicated increased off-odor for all groups through storage (Figure 5A, *p* < 0.001). The sensory panel significantly influenced the off-odor analysis (*p* < 0.001). Fish originating from ice or RSW gave a similar trend, regardless of the type of packaging material used after MAP. The RH and RP groups gave a better sensory score than the IH and IP groups (*p* < 0.001), in line with previous studies on sub-chilling. An interaction effect was observed between the chilling method and the packaging tray (*p* = 0.048). Sørensen, et al. [47] studied the effect of sub-chilled storage at −1.7 °C in combination with MAP (40% CO_2_:60% N_2_) on Atlantic cod and reported a sensory shelf life of more than 32 days. Moreover, Mei et al. [48] demonstrated that turbot in MAP (60% CO_2_:30% N_2_:10% O_2_) stored at sub-chilled conditions (−1.3 °C) maintained its freshness and prolonged the shelf life.

The microbiological growth on day 0 was under detection limits (2 log cfu/g for TPC and 1 log cfu/g for TMC and HSPB), indicating good initial microbial quality. Microbial growth increased through storage for all groups (Figure 5B–D; TPC: *p* < 0.001; TMC: *p* < 0.001; HSPB: *p* < 0.001). An aerobic count of >6 log cfu/g is considered spoiled [49,50]. Therefore, IP and IH were already spoiled after day 14. In contrast, RP and RH gave a longer microbiological shelf life when stored at sub-chilled conditions and were deemed spoiled after 49 days. Packaging in HDPE resulted in an increase in HSPB production as compared to the CPET trays (*p* = 0.006). In addition, the psychrotropic counts on L&H agar were higher than the total mesophilic counts on Iron agar in all groups. This has also been observed by Jääskeläinen et al. [51] on salmon, with *Photobacterium* as the dominating species.

#### Culture Independent Microbial Diversity

**Figure 6:**
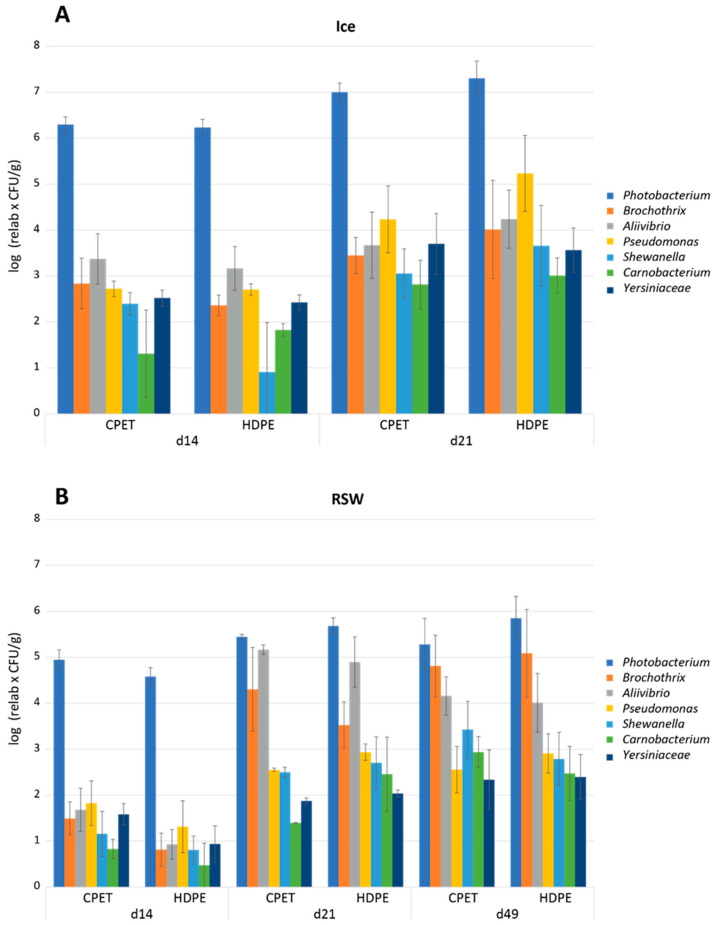
Estimated bacterial counts of the dominating (average above 0.02% across all samples) taxa (*Photobacterium*, *Brochothrix*, *Aliivibrio*, *Pseudomonas*, *Shewanella*, *Carnobacterium,* and *Yersiniaceae*) of (**A**) ice-stored fish and (**B**) RSW-stored fish in their respective packaging trays (CPET and HDPE) during storage (14, 21, and 49 days). The average of parallels (2–5) for the different packaging trays is represented with the standard deviation. The values are based on the relative amounts (%) from the Illumina partial 16S rRNA gene sequencing and the total aerobic psychotropic counts of bacteria in the samples (log_10_ (relative values × CFU/g)).

A total of 60 sOTUs (closely related bacterial sequences with single nucleotide differences) were detected from a total of 4,883,122 sequences after filtering (mean 113,561; min 65,642; max 159,274). The mean number of sequences per sOTU was 81,385 (max 4,440,814 and min 10). The microbiota of all fish samples were dominated by *Photobacterium* after 14 days of storage, with a relative abundance of 99.7% and 99.6% in fish originating from ice and RSW before MAP, respectively. The different packaging trays did not influence the microbiota. After 21 days of storage, fish originating from ice were still dominated by *Photobacterium* (98.6%), while the relative abundance of *Photobacterium* declined to 70.7% in fish originating from RSW due to the growth of *Allivibrio* (24.9%) and *Brochothrix* (3.8%). After 49 days of storage, the microbiota of fish originating from RSW had shifted to 68.2% *Photobacterium*, 24% *Brochothrix,* and 6.2% *Allivibrio*. The dominance of *Photobacterium* spp. confirmed results from other studies using CO_2_-modified atmospheres. *Photobacterium* is relatively resistant to CO_2_ [52,53,54] and has previously been found to dominate in MAP-stored fish [53,54] and also aerobically stored, head-on, gutted salmon [53] and cod fillets [54]. The bacterium has also been identified in seawater and live fish, which means cross-contamination of fillets is possible [55]. To better estimate microbial development during storage, the log of the relative abundance values multiplied by the total aerobic psychotropic counts was calculated. Figure 6 shows the estimated bacterial counts (average of 2–5 parallels) based on this calculation for fish originating from ice (A) and RSW (B).

Figure 6 shows that although *Photobacterium* is the dominating bacteria in fish stored in ice and RSW, the levels are much lower in fish originating from RSW than ice. This suggests that pre-storing at a lower temperature significantly increases the shelf life by delaying the bacterial growth (Figure 4B,C). However, further MAP storage results in the same dominating bacteria. That said, the levels of other bacteria were higher in fish originating from RSW than in ice. The second most abundant bacteria after 21 days of storage in fish originating from ice was *Pseudomonas*; however, the levels were approx. 2 log lower than *Photobacterium*. Fish originating from RSW had high levels of *Allivibrio* relative to *Photobacterium* after 21 days of storage, and of *Brochothrix* after 49 days of storage. High levels of *Photobacterium* will influence the sensory quality. *Photobacterium phosphoreum* is reported to be a specific spoilage organism (SSO) of MAP fish, related to their reduction of trimethylamine oxide (TMAO) to trimethylamine (TMA) [56,57,58,59,60]. The TMA production by *Photobacterium phosphoreum* is estimated to be 30 times higher compared to, e.g., Shewanella putrefaciens [59]. The genus *Aliivibrio* is composed of a few species (e.g., *A. fischeri*, *A. logei*, *A. salmonicida*), which were transferred from genus *Vibrio* to the newly created *Aliivibrio* genus in the family *Vibrionaceae* in 2007 [61]. The *Vibrionaceae* are predominately psychrotolerant and able to grow under MA, and they are also able to produce TMA [56]. *Brochothtix thermosphacta* is considered a potent spoilage bacterium in MAP shrimp and fish, and its growth is often boosted at the end of storage with other Gram-positive bacteria, especially lactic acid bacteria [62,63].

### 3.6. Lipid Oxidation

Around 93 volatile compounds were detected in the samples, and some of these were known lipid oxidation products. Among these, hexanal is a well-known volatile oxidation product from omega-6 fatty acids, while 1-penten-3-ol and 2,4- heptadienal are two well-known volatile oxidation products from omega-3 fatty acids that correlate well with sensory properties and rancidity in oils and food products [64]. The initial raw material contained a low level of 1-penten-3-ol (8 ppm), but no hexanal or 2,4-heptadienal were observed. After 49 days of sub-chilled storage, some oxidative changes were detected. The levels of 1-penten-3-ol increased and were higher in the RH group (79 ppm) than in the RP group (28 ppm). In addition, some increase in hexanal (RH: 4 ppm and RP: 3 ppm) and 2,4 heptadienal (RH: 2 ppm and RP: 2 ppm) were observed. The results aligned with the microbiology and sensory analyses, where the RH group had a higher psychrotrophic count and a higher off-odor score than the RP group.

## 4. Conclusions

This study established the hurdle concept of combining storage temperature and packaging methods. Sub-chilled storage significantly extended the sensory and microbiological shelf life of Atlantic salmon portions packaged in a modified atmosphere compared to storage at 4 °C. In addition, the sub-chilling process completely removes the use of ice, which carries significant weight and is commonly used to preserve fish in industries. Even though differences in OTR were detected between CPET and HDPE, almost no effect was detected on the quality parameters, demonstrating that mono-materials can be used, which would increase the recyclability of packaging materials. Combining sub-chilling and MAP can be carried out on a large scale, giving foods a longer shelf life and reducing food waste. However, designing the trays to fit the heights of the samples to avoid the top films from touching the samples is vital. Nevertheless, temperature stability during sub-chilling and sub-chilled storage is crucial to prevent temperature fluctuations that can negatively influence quality.

## Figures and Tables

**Table 1:** Colour and textural analyses of whole fish stored on ice and RSW until day 7 before further processing.

Day	Group	L*	a*	b*	Breaking Force (N)	Firmness (N)
0	Ice	66.6 ± 1.1	30.2 ± 2.0	28.1 ± 1.8	12.6 ± 3.2	24.7 ± 5.0
	RSW	66.6 ± 1.1	30.2 ± 2.0	28.1 ± 1.8	12.6 ± 3.2	24.7 ± 5.0
4	Ice	61.9 ± 2.1	33.0 ± 1.1	29.5 ± 1.2	6.1 ± 1.0	18.2 ± 4.6
	RSW	62.3 ± 1.4	33.1 ± 2.4	29.7 ± 2.7	7.2 ± 1.1	18.3 ± 3.0
7	Ice	67.1 ± 2.1	34.0 ± 2.9	31.3 ± 2.5	6.9 ± 0.6	17.8 ± 4.7
	RSW	66.0 ± 0.7	34.9 ± 1.3	31.0 ± 0.8	7.2 ± 1.2	17.6 ± 3.9
GLM ^a^	P_D_	<0.001 *	<0.001 *	0.005 *	<0.001 *	<0.001 *
	P_C_	0.668	0.613	0.943	0.458	1.000
	P_H_	-	-	-	0.002 *	0.442

^a^ General linear model (GLM) with group (ice, RSW) and storage days as factors. Fillet height was included as a covariate for statistical analysis. L*, a*, and b* represent lightness, redness, and yellowness, respectively. P_D_, P_C,_ and P_H_ represent the significant levels for the effects of storage days, chilling method, and fillet height, respectively. * Statistically significant when *p* < 0.05.

**Table 2:** Colour and textural analyses of packaged portions stored on IP (ice, CPET) and IH (ice, HDPE) for 21 days and RP (RSW, CPET) and RH (RSW, HDPE) for 56 days.

Day	Group	L*	a*	b*	Breaking Force (N)	Firmness (N)
11	IP	65.3 ± 1.3	32.8 ± 1.0	30.0 ± 0.8	6.9 ± 0.5	7.4 ± 1.0
	IH	65.9 ± 1.3	31.6 ± 1.3	28.7 ± 1.1	5.9 ± 1.0	7.2 ± 1.6
14	IP	65.8 ± 1.8	31.8 ± 1.1	28.5 ± 0.7	6.1 ± 1.2	8.2 ± 0.6
	IH	66.6 ± 1.7	32.1 ± 1.4	29.5 ± 1.3	6.0 ± 1.5	7.8 ± 1.2
	RP	65.5 ± 1.3	32.5 ± 1.0	29.4 ± 0.6	6.8 ± 0.9	8.5 ± 1.4
	RH	65.8 ± 1.3	31.9 ± 1.3	29.2 ± 1.2	5.1 ± 0.6	9.0 ± 1.5
18	IP	66.2 ± 1.7	31.7 ± 2.2	27.9 ± 1.6	6.0 ± 1.3	7.4 ± 1.6
	IH	67.4 ± 1.4	31.6 ± 1.9	27.9 ± 2.0	5.3 ± 1.1	6.8 ± 0.7
21	IP	63.9 ± 2.4	30.6 ± 2.9	27.3 ± 1.9	4.4 ± 1.1	5.8 ± 1.1
	IH	65.0 ± 2.2	29.9 ± 2.6	26.7 ± 1.3	4.6 ± 0.7	6.7 ± 1.3
	RP	62.7 ± 2.2	30.8 ± 1.2	27.9 ± 0.8	5.1 ± 1.2	6.9 ± 0.8
	RH	63.7 ± 2.3	30.2 ± 1.0	28.2 ± 1.6	4.7 ± 1.1	5.9 ± 1.5
29	RP	68.4 ± 1.6	32.4 ± 1.5	28.2 ± 0.9	5.6 ± 1.1	7.2 ± 1.3
	RH	68.2 ± 1.6	33.0 ± 1.5	30.3 ± 1.2	4.6 ± 0.7	7.1 ± 2.0
35	RP	66.3 ± 1.2	35.0 ± 3.6	31.4 ± 3.3	5.4 ± 1.6	5.3 ± 1.0
	RH	67.8 ± 1.9	31.1 ± 2.7	28.2 ± 1.7	4.6 ± 1.6	5.6 ± 1.5
42	RP	65.8 ± 0.6	34.0 ± 1.1	29.7 ± 1.4	4.4 ± 0.6	4.9 ± 0.9
	RH	67.8 ± 1.3	30.4 ± 2.7	28.4 ± 1.6	4.7 ± 1.2	6.1 ± 1.0
49	RP	66.1 ± 1.0	32.3 ± 1.4	29.5 ± 0.8	6.6 ± 1.7	7.5 ± 1.1
	RH	68.6 ± 1.7	28.6 ± 1.6	27.1 ± 0.8	5.3 ± 1.5	7.7 ± 0.7
56	RP	64.9 ± 1.7	33.3 ± 3.0	28.2 ± 3.1	4.5 ± 1.0	6.5 ± 1.2
	RH	66.5 ± 2.0	30.9 ± 2.4	27.1 ± 1.3	4.8 ± 1.1	7.4 ± 2.0
GLM ^a^	P_D_	<0.001 *	0.016 *	0.004 *	<0.001 *	<0.001 *
	P_C_	0.083	0.675	0.184	0.834	0.157
	P_P_	<0.001 *	<0.001 *	0.059	0.020 *	0.665
	P_H_	-	-	-	0.001 *	0.012 *

^a^ General linear model (GLM) with group (IP, IH, RP, RH) and storage days as factors. Fillet height was included as a covariate for statistical analysis. L*, a*, and b* represent lightness, redness, and yellowness, respectively. P_D_, P_C_, P_p,_ and P_H_ represent the significant levels for the effects of storage days, chilling method, packaging tray, and fillet height, respectively. * Statistically significant when *p* < 0.05.

## Data Availability

Data are contained within the article.
